# Supplementary material for: Outcomes for Patients Receiving Multi-Chamber Bags for the Delivery of Parenteral Nutrition: A Systematic Review
Source: Nutrients. 2024 Nov 20;16(22):3964. doi: 10.3390/nu16223964 (PMC11597339; doi:10.3390/nu16223964)
Supplement: Supplementary file 1 [file nutrients-16-03964-s001.zip › nutrients-3312706-supplementary.pdf]

Supplementary Information

Table S1: Prisma Checklist 2020

Section and Topic	Item	Checklist item	Page where item is reported
**TITLE**			
Title	1	Identify the report as a systematic review.	1
**ABSTRACT**			
Abstract	2	See the PRISMA 2020 for Abstracts checklist.	1
**INTRODUCTION**			
Rationale	3	Describe the rationale for the review in the context of existing knowledge.	2
Objectives	4	Provide an explicit statement of the objective(s) or question(s) the review addresses.	2
**METHODS**			
Eligibility criteria	5	Specify the inclusion and exclusion criteria for the review and how studies were grouped for the syntheses.	2
Information sources	6	Specify all databases, registers, websites, organisations, reference lists and other sources searched or consulted to identify studies. Specify the date when each source was last searched or consulted.	3
Search strategy	7	Present the full search strategies for all databases, registers and websites, including any filters and limits used.	3 and SI figure S1
Selection process	8	Specify the methods used to decide whether a study met the inclusion criteria of the review, including how many reviewers screened each record and each report retrieved, whether they worked independently, and if applicable, details of automation tools used in the process.	3
Data collection process	9	Specify the methods used to collect data from reports, including how many reviewers collected data from each report, whether they worked independently, any processes for obtaining or confirming data from study investigators, and if applicable, details of automation tools used in the process.	4
Data items	10a	List and define all outcomes for which data were sought. Specify whether all results that were compatible with each outcome domain in each study were sought (e.g. for all measures, time points, analyses), and if not, the methods used to decide which results to collect.	4
	10b	List and define all other variables for which data were sought (e.g. participant and intervention characteristics, funding sources). Describe any assumptions made about any missing or unclear information.	4
Study risk of bias assessment	11	Specify the methods used to assess risk of bias in the included studies, including details of the tool(s) used, how many reviewers assessed each study and whether they worked independently, and if applicable, details of automation tools used in the process.	4
Effect measures	12	Specify for each outcome the effect measure(s) (e.g. risk ratio, mean difference) used in the synthesis or presentation of results.	5
Synthesis methods	13a	Describe the processes used to decide which studies were eligible for each synthesis (e.g. tabulating the study intervention characteristics and comparing against the planned groups for each synthesis (item #5)).	5
	13b	Describe any methods required to prepare the data for presentation or synthesis, such as handling of missing summary statistics, or data conversions.	5
	13c	Describe any methods used to tabulate or visually display results of individual studies and syntheses.	5
	13d	Describe any methods used to synthesize results and provide a rationale for the choice(s). If meta-analysis was performed, describe the model(s), method(s) to identify the presence and extent of statistical heterogeneity, and software package(s) used.	5
	13e	Describe any methods used to explore possible causes of heterogeneity among study results (e.g. subgroup analysis, meta-regression).	5
	13f	Describe any sensitivity analyses conducted to assess robustness of the synthesized results.	5
Reporting bias assessment	14	Describe any methods used to assess risk of bias due to missing results in a synthesis (arising from reporting biases).	5
Certainty assessment	15	Describe any methods used to assess certainty (or confidence) in the body of evidence for an outcome.	5
**RESULTS**			
Study selection	16a	Describe the results of the search and selection process, from the number of records identified in the search to the number of studies included in the review, ideally using a	5 and Figure 1

Section and Topic	Item	Checklist item	Page where item is reported
		flow diagram.	
	16b	Cite studies that might appear to meet the inclusion criteria, but which were excluded, and explain why they were excluded.	5
Study characteristics	17	Cite each included study and present its characteristics.	6 - 8 and Tables 1 & 2
Risk of bias in studies	18	Present assessments of risk of bias for each included study.	10 and SI Tables S4-S6
Results of individual studies	19	For all outcomes, present, for each study: (a) summary statistics for each group (where appropriate) and (b) an effect estimate and its precision (e.g. confidence/credible interval), ideally using structured tables or plots.	12-18 and Tables 3-5
Results of syntheses	20a	For each synthesis, briefly summarise the characteristics and risk of bias among contributing studies.	12-18
	20b	Present results of all statistical syntheses conducted. If meta-analysis was done, present for each the summary estimate and its precision (e.g. confidence/credible interval) and measures of statistical heterogeneity. If comparing groups, describe the direction of the effect.	12-18
	20c	Present results of all investigations of possible causes of heterogeneity among study results.	12-18
	20d	Present results of all sensitivity analyses conducted to assess the robustness of the synthesized results.	12-18
Reporting biases	21	Present assessments of risk of bias due to missing results (arising from reporting biases) for each synthesis assessed.	12-18
Certainty of evidence	22	Present assessments of certainty (or confidence) in the body of evidence for each outcome assessed.	12-18
**DISCUSSION**			
Discussion	23a	Provide a general interpretation of the results in the context of other evidence.	18-19
	23b	Discuss any limitations of the evidence included in the review.	19
	23c	Discuss any limitations of the review processes used.	19
	23d	Discuss implications of the results for practice, policy, and future research.	19
**OTHER INFORMATION**			
Registration and protocol	24a	Provide registration information for the review, including register name and registration number, or state that the review was not registered.	20
	24b	Indicate where the review protocol can be accessed, or state that a protocol was not prepared.	20
	24c	Describe and explain any amendments to information provided at registration or in the protocol.	20
Support	25	Describe sources of financial or non-financial support for the review, and the role of the funders or sponsors in the review.	20
Competing interests	26	Declare any competing interests of review authors.	20
Availability of data, code and other materials	27	Report which of the following are publicly available and where they can be found: template data collection forms; data extracted from included studies; data used for all analyses; analytic code; any other materials used in the review.	Tables 1-5, Tables S2-S6

Table S2: Inclusion and exclusion criteria for screening articles

The aim of this systematic review is to evaluate the literature on the effect of people receiving hospital or home PN in the format of multichamber bags on clinical outcomes and quality of life

	**Inclusion criteria**	**Exclusion criteria**
**Population**<br>Age<br><br><br><br>Disease	18 years and older (also include studies if more than 80% of participants are over 18. If the age of the patients is not specified it will be assumed that the patients are adults).<br><br>Human population receiving parenteral nutrition	• Studies with individuals all under the age of 18.<br>• Studies in animals.
**Intervention**	Interventions or exposures that included the provision of standardised PN delivered using MCBs.	Studies or data sets within studies where patients were receiving intravenous fluids and electrolytes only with no macronutrients.
**Comparators**	• PN delivered using individually compounded bags<br>• studies that considered clinical outcomes and patient acceptance of MCBs without a comparator.	
**Outcomes**<br>Primary<br><br><br>Secondary	• Whether participants met their nutritional requirements and maintained their weight Food group intake<br><br>• Number and type of complications<br>• Patients experience (including quality of life)<br>• Cost	
**Settings**	Home and hospital settings	
**Date and language**	Articles published from 2015 up to present day. No restrictions on language.	
**Study design**	• RCTs (including cluster RCTs)<br>• Controlled (non-randomized) clinical trials<br>• Cluster trials<br>• Prospective and retrospective comparative cohort studies<br>• Cross-sectional studies<br>• Qualitative studies using grounded theory<br>• Phenomenology thematic analysis<br>• Case studies, and case-control studies (with at least 5 or more cases)	• Case and case-control studies with less than 5 cases.

Table S3: Terminology used in the international literature for bag types

Multichamber	Compounded	Ambiguous
MCB<br>Multi-chamber bag<br>Commercial ready to use<br>3 chamber bag/3-chamber bag<br>3CBs/3-CBs<br>2 chamber bag/2-CB<br>3 in 1/3-in-1<br>2 in 1/2-in-1<br>3 compartment bag/3-compartment bag<br>2 compartment bag/2-compartment bag<br>Triple chamber bag/triple-chamber bag<br>Dual chamber bag/dual-chamber bag	COM<br>Tailored<br>Tailor-made<br>Bespoke<br>Individualised/Individualized<br>Customised/Customized<br>Hospital-compounded<br>Pharmacy compounded<br>COB	[a]All-in-one/All in one/AIO<br>[b]Premixed/Pre-mixed<br>Premade/Pre-made<br>[b]Ready to use/ready-to-use<br>Commercially pre-mixed<br>[c]Admixtures/Industrial admixtures

[a]All in one: in some cases to refer to compounded bag as the bag containing the solution is a single, large chamber. In other cases it to refer to a bag with a single hang point (as opposed to a multi bottle system) which could therefore indicate either a multichamber bag or compounded bag.
[b]Premixed or ready to use could potentially refer either to compounded bags (i.e., all 3 macronutrients premixed into one bag and ready to hang) or to a multichamber bag, the chambers of which had been 'rolled' in pharmacy to separate the intrachamber seals to mix all three into one.
[c]Industrial admixtures: This term often refers to multichamber bags. Some commercial pharmaceutical companies do prepare PN bags individualised to a single patient's requirements, according to the prescription provided by the clinical team. The term could therefore be ambiguous and not recommended.

Figure S1: Search strategies for all databases and trial registries

Medline(R) 1946 to October Week 4 2022 (Ovid Platform)

1. Exp Parenteral Nutrition/
2. exp Parenteral Nutrition Solutions/
3. (parenteral* adj3 (nutri* or hydration* or feed* or fed* or treatment* or manag* or method* or car* or support* or diet*)).mp.
4. (nutrition adj5 (venous line* or central line* or Hickman line*)).mp.
5. ((parenteral or intravenous) adj5 (nutrition or feeding)).mp.
6. (parenteral$ adj2 (fed or feed$)).ti,ab.
7. total parenteral nutrition*.mp.
8. TPN.mp.
9. PN.mp.
10. parenteral nutrition*.mp.
11. (3-chamber* bag* or 3 chamber* bag* or 3-compartment premixed or 3-compartment or 3CB or 3-in-1 PN or three-compartment bag*).mp.
12. (Triple chamber* or Triple-chamber*).mp.
13. (2 chamber* bag* or 2-chamber* bag* or 2-compartment premixed or 2-in-1 PN or two-compartment bag).mp.
14. (multi-bottle system or multibottle).mp.
15. (Multichamber* bag* or multi-chamber* bag*or multi chamber* bag* or multi-compartment* bag* or multi-chamber*).mp.
16. industrial preparations.mp.

17. (MCB or MCB*).mp.
18. all-in-one.mp
19. (compound* bag* or pharmacy-compounded or tailor-made or individuali#ed admixtures) .mp
20. 1 or 2 or 3 or 4 or 5 or 6 or 7 or 8 or 9 or 10
21. 11 or 12 or 13 or 14 or 15 or 16 or 17 or 18 or 19
22. 20 and 21
23. limit 22 to yr="2015 -Current"

Embase 1974 to 2022 November 01 (Ovid platform)

1. Exp Parenteral Nutrition/
2. (parenteral* adj3 (nutri* or hydration* or feed* or fed* or treatment* or manag* or method* or car* or support* or diet* or home)).mp.
3. (nutrition adj5 (venous line* or central line* or Hickman line*)).mp.
4. ((parenteral or intravenous) adj5 (nutrition or feeding)).mp.
5. (parenteral$ adj2 (fed or feed$)).ti,ab.
6. total parenteral nutrition*.mp.
7. TPN.mp.
8. PN.mp.
9. parenteral nutrition*.mp.
10. (3-chamber* bag* or 3 chamber* bag* or 3-compartment premixed or 3-compartment or 3CB or 3-in-1 PN or three-compartment bag*).mp.
11. (Triple chamber* or Triple-chamber*).mp.
12. (2 chamber* bag* or 2-chamber* bag* or 2-compartment premixed or 2-in-1 PN or two-compartment bag).mp.
13. (multi-bottle system or multibottle).mp.
14. (Multichamber* bag* or multi-chamber* bag*or multi chamber* bag* or multi-compartment* bag* or multi-chamber*).mp.
15. industrial preparations.mp.
16. (MCB or MCB*).mp.
17. all-in-one.mp
18. (compound* bag* or pharmacy-compounded or tailor-made or individuali#ed admixtures) .mp
19. 1 or 2 or 3 or 4 or 5 or 6 or 7 or 8 or 9
20. 10 or 11 or 12 or 13 or 14 or 15 or 16 or 17 or 18
21. 19 and 20
22. limit 21 to yr="2015 -Current"

PsycInfo 1806 to October Week 4 2022 (Ovid Platform)

1. exp Parenteral Nutrition/
2. exp Parenteral Nutrition Solutions/
3. (parenteral* adj3 (nutri* or hydration* or feed* or fed* or treatment* or manag* or method* or car* or support* or diet* or home)).mp.

4. (nutrition adj5 (venous line* or central line* or Hickman line*)).mp.
5. ((parenteral or intravenous) adj5 (nutrition or feeding)).mp.
6. (parenteral$ adj2 (fed or feed$)).ti,ab.
7. total parenteral nutrition*.mp.
8. TPN.mp.
9. PN.mp.
10. parenteral nutrition*.mp.
11. (3-chamber* bag* or 3 chamber* bag* or 3-compartment premixed or 3-compartment or 3CB or 3-in-1 PN or three-compartment bag*).mp.
12. (Triple chamber* or Triple-chamber*).mp.
13. (2 chamber* bag* or 2-chamber* bag* or 2-compartment premixed or 2-in-1 PN or two-compartment bag).mp.
14. (multi-bottle system or multibottle).mp.
15. (Multichamber* bag* or multi-chamber* bag*or multi chamber* bag* or multi-compartment* bag* or multi-chamber*).mp.
16. industrial preparations.mp.
17. (MCB or MCB*).mp.
18. all-in-one.mp
19. (compound* bag* or pharmacy-compounded or tailor-made or individuali#ed admixtures) .mp
20. 1 or 2 or 3 or 4 or 5 or 6 or 7 or 8 or 9 or 10
21. 11 or 12 or 13 or 14 or 15 or 16 or 17 or 18 or 19
22. 20 and 21
23. limit 22 to yr="2015 -Current"

Cochrane Central Registry of Controlled Trials

Cochrane library publication date from Jan 2015 to present

1. MeSH descriptor: [Parenteral Nutrition] 1 tree(s) exploded
2. MeSH descriptor: [Parenteral Nutrition Solutions] explode all trees
3. parenteral* NEAR/3 (nutri* or hydration* or feed* or fed* or manag* or method* or car* or support* or diet* or home)
4. nutrition NEAR/5 ("venous line*" or "central line*" or "Hickman line*")
5. (parenteral or intravenous) NEAR/5 (nutrition or feeding)
6. (parenteral* NEAR/2 (fed or feed?)
7. "total parenteral nutrition*"
8. TPN
9. PN
10. "parenteral nutrition"
11. "3-chamber* bag*" or "3 compartment" or "3 compartment bag*" 3CB or "3-in-1"
12. "Triple chamber*"

13. “2 chamber* bag*” or “2 compartment” or “two compartment bag*” or 2CB or “2 in 1” or “dual chamber*”
14. “multibottle system” or multibottle
15. “Multichamber* bag*” or “multi chamber* bag*” or “multi compartment* bag*” or “multi chamber*”
16. “industrial preparations”
17. (MCB or MCB*)
18. “all in one”
19. “compound* bag*” or “pharmacy compounded” or “tailor made” or “individuali?ed admixtures” or bespoke
20. #1 or #2 or #3 or #4 or #5 or #6 or #7 or #8 or #9 or #10
21. #11 or #12 or #13 or #14 or #15 or #16 or #17 or #18 or #19
22. #20 and #21

CINAHL Plus (EBSCOhost platform)

Applied to all lines:

- Expanders: Apply equivalent subjects
- Search modes: Boolean/Phrase

Applied to lines 20 and 21:

- Published date: 20150101-20221131

1. (MH “Parenteral Nutrition+”)
2. (MH “Parenteral Nutrition Solutions”)
3. (parenteral* N3 (nutri* or hydration* or feed* or fed* or treatment* or manag* or method* or car* or support* or diet* or home))
4. (nutrition N5 (“venous line*” or “central line*” or “Hickman line*”)
5. (parenteral or intravenous) N5 (nutrition or feeding)
6. (parenteral* N5 (fed or feed*)ti.ab.
7. “total parenteral nutrition*”
8. TPN
9. PN
10. “parenteral nutrition*”
11. “3-chamber* bag*” or “3 compartment*” or “3 compartment bag” or 3CB* or “3 in 1” or “three chamber bag*” or “three compartment*” or “three compartment bag*” or “three in one”
12. “Triple chamber*”
13. “2 chamber* bag*” or “2 compartment*” or “2 compartment bag” or 2CB* or “2 in 1” or “two chamber* bag*” or “two compartment*” or “two compartment bag*” or “two in one” or “dual chamber”
14. “multibottle system” or multibottle
15. “Multichamber* bag*” or “multi-chamber* bag*” or "multi compartment* bag*” or “multicompartment* bag*” or multichamber*)
16. “industrial preparations”

17. MCB or MCB*
18. “all in one”
19. “compound* bag*” or “pharmacy compounded” or “tailor made” or “individuali?ed admixtures” or bespoke
20. S1 or S2 or S3 or S4 or S5 or S6 or S7 or S8 or S9 or S10
21. S11 or S12 or S13 or S14 or S15 or S16 or S17 or S18 or S19
22. S20 and S21

Web of Science (Clarivate platform)

1. ALL=(“Parenteral Nutrition”)
2. ALL=(“Parenteral Nutrition Solutions”)
3. TS=(parenteral* NEAR/2 (nutri* or hydration* or feed* or fed* or manag* or method* or car* or support* or diet* or home))
4. TS=(nutrition NEAR/4 (“venous line” or “central line” or “Hickman line”))
5. TS=((parenteral or intravenous) NEAR/4 (nutrition or feeding))
6. TS=(parenteral* NEAR/1 (fed or feed*))
7. AK=(“total parenteral nutrition”)
8. AK=(“TPN”)
9. AK=(“PN”)
10. AK=(“parenteral nutrition”)
11. AK=(“3 chamber* bag*” or “3 compartment” or “3 compartment bag*” 3CB or “3 in 1” or “three chamber* bag*” or “three compartment*” or “three compartment bag*” or “three in one”)
12. AK=(“Triple chamber*”)
13. AK=(“2 chamber* bag*” or “2 compartment” or “two compartment bag*” or 2CB or “2 in 1” or “dual chamber” or “two chamber* bag*” or “two compartment” or “two compartment bag*” or “two in one”)
14. AK=(“multibottle system” or multibottle)
15. AK=(“Multichamber bag*” or “multi chamber* bag*” or “multi compartment* bag*” or “multi chamber*”)
16. AK=(“industrial preparations”)
17. AK=(“MCB” or “MCB*”)
18. AK=(“all in one”)
19. AK=(“compound* bag*” or “pharmacy compounded” or “tailor made” or “individuali?ed admixtures”)
20. #1 or #2 or #3 or #4 or #5 or #6 or #7 or #8 or #9 or #10
21. #11 or #12 or #13 or #14 or #15 or #16 or #17 or #18 or #19
22. #20 and #21
23. #20 and #21 (limit to yr=“2015 -Current”)

Tables S4 to S6: Full Joanna Briggs Quality Assessments

Table S4: Cohort studies

Author	Q1	Q2	Q3	Q4	Q5	Q6	Q7	Q8	Q9	Q10	Q11	Overall quality
Crooks, 2022	Y	Y	Y	Y	Y	Y	Y	Y	U	U	Y	High*
	1) Patients with Chronic IF receiving HPN at a national UK referral centre. 2 & 3) Confirmed diagnosis of CIF and receiving MCB on customised PN. 4) Potential confounders included the catheter care provider, cause of CIF, CVC type, and mean daily volume of IV supplementation. 5) Multifactorial binary logistical regression was used to adjust for confounders. 6) Outcome is catheter related infections, so can only occur whilst being exposed. 7) Diagnosis of catheter related bloodstream infection was based on clinical signs of sepsis in tandem with quantitative and/or qualitative analysis of paired central and peripheral blood cultures. 8) 14 months follow up with incidence described per 1000 catheter days. 9 & 10) Not reported. 11) Student t-test was used for continuous variables and chi-square test for categorical variables. Multifactorial binary logistical regression was used to adjust for confounders.											
Fernández-Argüeso, 2024	Y	Y	Y	Y	Y	Y	Y	Y	Y	N/A	Y	High
	1) Patients from oncology ward with advanced cancer and amenable to anti-cancer therapy. Similar age and gender across groups. 2&3) Patients received either compounded or MCB, or both, during their follow-up, as required. The choice of the type of bag was not randomised but was indicated by the patients' needs and the characteristics of the compounded bags. After hospital discharge, HPN was infused on an intermittent schedule, primarily at nighttime. 4&5) multivariate analysis with cox proportional hazards test performed. 6,7,8) Yes as outcomes relate to factors occurring later in the process (complications). 9) followed from 2007 to 2002 and incidence recorded until weaned off HPN, death or end of study. 10) N/A. 11) yes, Logarithmic or square root transformations were applied as needed, to ensure a normal distribution of the variables. Comparisons between the different groups at baseline were performed by independent t test for continuous variables or the Mann–Whitney U test for non-normal distributed variables, and by χ 2 test or Fisher's exact test for discontinuous variables. Survival was analysed by Kaplan–Meier estimator, and the log rank test and multivariate Cox proportional hazards test and hazard ratios (HR) calculated											
Goh, 2022	U	Y	Y	N	N	U	N	N	N	N	Y	Low*
	1) Patients starting on PN in the general wards of Singapore General Hospital. Patients in compounded group lower BMI due to patients with low BMI not been given standard bags so possibly more malnourished group 2 & 3) Standardized commercially available PN product or compounded PN. 4 & 5) Confounding factors not discussed and no strategies stated. 6) Outcomes were data collected on nutritional intake. 7&8) Follow up time was only 1 day and the main outcome was to assess safety of bridging from standardized to customised, so may not be appropriate to compare one to the other over such a short time period when the aim is to use standardized PN as a 'bridge'. 9 &10) not stated and assume this is due to study being carried out over 24 hours only. 11) The Student's t-test was used for continuous variables and Fisher's exact test used for categorical data.											
Zhao, 2018	Y	Y	Y	N	N	N/A	U	Y	Y	N/A	Y	Medium*
	1) Gastric cancer patients who underwent gastrectomy from 2014 to 2016 in a tertiary teaching hospital in Beijing, China. Table 1 shows no differences at baseline (demographics, BMI, bloods) under p of 0.05. However, differences in gender between the two groups were borderline at p = 0.057. 2 & 3) Patients were categorized into standardized (s-TPN) and customized TPN (c-TPN) groups based on their TPN order after gastrectomy. 4 & 5) "We excluded pts whose regimen did not comply with CSPEN guidelines to control for confounding."- This would not necessarily effectively control confounding. Complexity of prescription in electrolytes was likely to have been a confounder that was not identified as "patients were categorized into standardized and customized TPN based on their TPN order" "s-TPN received higher levels of aas, fat emulsions, Ca, Mg and PO4" Also fat emulsions were different. 6) Outcomes were effectiveness, cost and safety related so not applicable. 7) Outcomes relating to the patient were gathered from medical charts. For cost the calculation appears to have failed to include production costs. 8) Followed patients from the first day of postoperative parenteral nutrition (PN) support to discharge. 9) study was retrospective and all patients who were included were followed until discharge. 10) follow up was complete. 11) Paired t-test (if normally distributed) or Wilcoxon signed ranks test (if not normally distributed) was used to make within group comparisons. Independent t-test (if normally distributed) or Mann-Whitney U test (if not normally distributed) was used to do between group comparisons.											
Xie, 2018	Y	U	U	N	N	Y	Y	U	U	U	Y	Low*

	1) No significant difference between number, gender and age of participants between groups. Whether there was a difference in cancer site and type is not stated. 2) The full nutritional content of the MCB and COM regimens is not given. It appears compounded had lipid as MCT and LCT. Lipid type for MCB not stated. Calories, protein infused not stated. 3) "Clinical data of 334 patients undergoing gastrointestinal tumour surgery in the Second Hospital of Jilin University from January 1, 2016 to December 31, 2016 were collected" According to the formulas used by patients for nutritional support, participants were divided into COM group and MCB group. 4&5) None stated. 6&7) Yes as most outcomes relate to factors occurring later in the process – e.g. length of stay, re-operation rate. Unclear whether for infections, pts with infection prior to starting PN were identified, and the data adjusted appropriately. 8, 9, 10) Not reported. 11) SPSS 20.0 was used for statistical analysis of the data. The t-test was used for measurement data, and the χ2 test was used for the comparison of count data.

N= No, Y = Yes, U= unclear, N/A = Not applicable, CIF = Chronic Intestinal Failure, PN = Parenteral Nutrition, HPN = Home parenteral nutrition, TPN = Total parenteral Nutrition, MCB = Multi chamber bag.
**Retrospective study design and so quality can be further degraded from what is stated due to potential selection bias.*
Questions: 1) Were the two groups similar and recruited from the same population?, 2) Were the exposures measured similarly to assign people to both exposed and unexposed groups? 3) Was the exposure measured in a valid and reliable way? 4) Were confounding factors identified? 5) Were strategies to deal with confounding factors stated? 6) Were the groups/participants free of the outcome at the start of the study (or at the moment of exposure)? 7) Were the outcomes measured in a valid and reliable way? 8) Was the follow up time reported and sufficient to be long enough for outcomes to occur? 9) Was follow up complete, and if not, were the reasons to loss to follow up described and explored? 10) Were strategies to address incomplete follow up utilized? 11) Was appropriate statistical analysis used?

Table S5: Cross sectional studies

Author	Q1	Q2	Q3	Q4	Q5	Q6	Q7	Q8	Overall quality
Banko, 2019	Y	Y	Y	Y	Y	Y	Y	Y	High*
	1 &2) Retrospective study of blood stream infections and cost for discharged inpatients on MCB PN no additions, MCB PN with additions and compound PN in hospital - data taken from Premier Healthcare Database - largest hospital discharge database in US. Inclusion and exclusion criteria given and information on patient characteristics and hospitals 3&4) Time of exposure was recorded and it was clear which type of PN patients were having. 5&6) Carried out multivariable regression modelling for all potential confounders including patient, hospital and clinical characteristics. The Hosmer and Lemeshow goodness-of-fit test was used to assess the fit of each model. Identified that more patients on MCB were admitted as emergencies this was adjusted for in the multiple regression 7) blood stream infections identified using diagnostic codes and costs via consumer hospital index – no allowances though for staff time and indirect costs 8) Descriptive stats were used to describe patients within each preparation method, Chi square tests were used to examine statistical differences in categorical variables. Different type of multiple regression modelling used for different outcomes.								
Park, 2020	Y	Y	Y	Y	Y	N	N	N	Medium*
	1 & 2) Recruitment from 9 Korean hospitals with memberships to Korean pharmacy and PN societies. Hospitalised patients receiving PN between on 1st Aug 2017 – 30th Oct 2017. Patients who were pregnant or less than 19 years old were excluded. 3 & 4) Type of PN administration, formulation and time period of administration were all recorded. 5 & 6) Recorded potential confounding factors but did not use any strategies to deal with these in analysis.7 & 8) Outcomes were recorded as frequencies and occurrences and were not compared back to type of PN administration. Statistical analysis was only descriptive in nature.								

N= No, Y = Yes, PN = Parenteral nutrition, MCB Multi-chamber bag.
**Retrospective study design and so quality can be further degraded from what is stated due to potential selection bias.*
Questions: 1) Were the criteria for inclusion in the sample clearly defined?, 2) Were the study subjects and the setting described in detail?, 3) Was the exposure measured in a valid and reliable way?, 4) Were objective, standard criteria used for measurement of the condition?, 5) Were confounding factors identified?, 6) Were strategies to deal with confounding factors stated?, 7) Were the outcomes measured in a valid and reliable way?, 8) Was appropriate statistical analysis used?

Table S6: Randomised controlled trials

Author	Q1	Q2	Q3	Q4	Q5	Q6	Q7	Q8	Q9	Q10	Q11	Q12	Q13	Overall quality

<table>
<tr><td rowspan="2">Jia, 2015</td><td>Y</td><td>Y</td><td>Y</td><td>U</td><td>N</td><td>Y</td><td>N</td><td>Y</td><td>Y</td><td>U</td><td>U</td><td>Y</td><td>Y</td><td>Medium</td></tr>
<tr><td colspan="14">1&2) A true randomisation process was used and the allocation of participants was concealed from treatment group allocators. 3) the demographic and baseline clinical characteristics of patients in the olive oil and the soybean groups (ITT population) were not different. 4) Treatment assignment was not known by data management, biostatistical and personal at the central lab. However, doesn’t state whether patients knew which treatment they received, but it is possible they knew as they would be able to see the bag/lift any opaque cover to view. 5) It was not feasible to conduct a blinded study because it is standard practice that the physician and/or nurse carefully inspect the PN formulations to ensure integrity is maintained throughout infusion. 6) data management, biostatistical and personal at the central lab were blinded to treatment assignment. 7)Intervention of interest is MCB vs Compounded. MCB had olive oil PN and Compounded had soybean oil, so not treated identically. 8) Analyses were completed on intention to treat, modified intention to treat and per protocol population. 9) Participants are analysed in their randomised groups (olive oil or soy bean) regardless of this being ITT, mITT or PP. 10&11) Not enough detailed explanation for how outcomes are measured. 12) Complex and appropriate analysis undertaken. 13) This is a well-designed trial with true randomisation, large sample size, and analysed taking into account ITT, mITT and PP populations.</td></tr>
<tr><td rowspan="2">Xi, 2021</td><td>Y</td><td>Y</td><td>Y</td><td>U</td><td>U</td><td>U</td><td>U</td><td>Y</td><td>Y</td><td>U</td><td>U</td><td>U</td><td>Y</td><td>Medium</td></tr>
<tr><td colspan="14">1&2) A true randomisation process was used and the allocation of participants was concealed from treatment group allocators. 3) no statistically significant difference between the two groups in gender, age, height, weight, BMI and other demographic characteristics, disease and nutritional status. 4&5) unclear. 6) Not stated. 7) Stated that difference in surgical treatment taken into account in analysis, but no regression. 8) Among 240 patients, 235 were included in the full analysis set (FAS) analysis (experimental/ test group 120, control group 115), accounting for 97.92% of enrolled participants (experimental group 98.36%, control group 97.46%). 9) Appeared to stay in the same treatment groups. 10&11) Not reported. 12) Appropriate but unclear how this adjusted without regression.13) RCT appears appropriate. Design appears appropriate for comparison of prep time and biochemical/safety results.</td></tr>
<tr><td rowspan="2">Yu, 2017</td><td>Y</td><td>Y</td><td>Y</td><td>Y</td><td>U</td><td>N</td><td>N</td><td>Y</td><td>Y</td><td>U</td><td>U</td><td>Y</td><td>Y</td><td>Medium</td></tr>
<tr><td colspan="14">1, 2 & 4) A true randomisation process was used and the allocation of participants was concealed from treatment group allocators. Infusion bags were covered with an opaque bag and labelled with the randomization number to conceal from participants. 3) Assessment of nutrition efficacy of study and control regimens was restricted to normal-weight patients undergoing abdominal surgery. 5) Bags were covered did not state anything about the type of connector port. 6) Can reasonably assume lab staff were blinded in performing blood tests, but that assessors timing prep time could not have been. 7) No, The MCB had group received a mix of 50% MCT and 50% LCT whereas compounded group received 100% LCT lipid emulsion. 8&9) 6 patients lost in study group and 13 patients lost in control group giving a full analysis set of 115 in study group and 105 in control. Safety analysis used full ITT sets. Other stats were performed on both the PP Set and Full Analysis Set. 10&11) Lab parameters were determined on post op days 1 and 7 at the lab of each centre, although no. of centres and their procedures not stated. No details given as to comparability of how prep time was measured. 12) appropriate: The primary variable was analysed using paired t test and analysis of covariance (ANCOVA) to compare the variance of prealbumin level on pre op day 7 and its variance relative to baseline. 13) Overall trial design is appropriate but there were some limitations, which the authors included in the discussion. Main design flaw was: difference in lipid composition between the MCB and compounded group.</td></tr>
</table>

ITT: intension to treat, mITT: modified intention to treat, PP: per protocol, PN: parenteral nutrition, MCB: multichamber bag.

Questions: 1) Was true randomisation used for assignment of participants to treatment groups? 2) Was allocation to treatment groups concealed? 3) Were treatment groups similar at baseline? 4) Were participants blind to treatment assignment? 5) Were those delivering treatment blind to treatment assignment? 6) Were outcome assessors blind to treatment assignment? 7) Were the treatment groups treated identically other than the intervention of interest? 8) Was follow up complete and if not, were differences between groups in terms of their follow up adequately described and analysed? 9) Were participants analysed in the groups to which they were randomised? 10) Were outcomes measured in the same way for treatment groups? 11) Were outcomes measured in a reliable way? 12) Was appropriate statistical analysis used? 13) Was the trial design appropriate and any deviations from the standard RCT design (individual randomisation, parallel groups) accounted for in the conduct and analysis of the trial?
